# Pedodontic Considerations in a Child with Attention Deficit Hyperactivity Disorder: Literature Review and a Case Report

**DOI:** 10.5005/jp-journals-10005-1522

**Published:** 2018-06-01

**Authors:** Siddhi Sinha, Prasanna Praveen, S Prathibha Rani, Athimuthu Anantharaj

**Affiliations:** 1 Postgraduate Student, Department of Pedodontics and Preventive Dentistry, DA Pandu Memorial RV Dental College and Hospital, Bengaluru Karnataka, India; 2 Professor and Head, Department of Pedodontics and Preventive Dentistry, DA Pandu Memorial RV Dental College and Hospital, Bengaluru Karnataka, India; 3 Reader, Department of Pedodontics and Preventive Dentistry, DA Pandu Memorial RV Dental College and Hospital, Bengaluru Karnataka, India; 4 Professor, Department of Pedodontics and Preventive Dentistry, DA Pandu Memorial RV Dental College and Hospital, Bengaluru Karnataka, India

**Keywords:** Attention deficit hyperactivity disorder, Behavior, Dental management, Pediatric patient.

## Abstract

Attention deficit hyperactivity disorder (ADHD) is a common psychiatric disorder, characterized by extremely short attention span, impulsivity with resultant behavioral problems. They are prone to trauma and frequent dental injuries. The behavioral issues, cognitive deficits, and short attention span pose a challenge to the dental health team. Management of these children requires several modifications in approach at all levels of prevention and treatment. This study reviews all the pertinent oral health issues in these children and also presents the case report of a 14-year-old boy diagnosed with ADHD.

**How to cite this article:** Sinha S, Praveen P, Rani SP, Anantharaj A. Pedodontic Considerations in a Child with Attention Deficit Hyperactivity Disorder: Literature Review and a Case Report. Int J Clin Pediatr Dent 2018;11(3):254-259.

## INTRODUCTION

The Diagnostic and Statistical Manual of Mental Disorders, 5th Edition (DSM-V™) defines ADHD as a persistent pattern of inattention and/or hyperactivity-impulsivity that interferes with functioning or development, has symptoms presenting in two or more settings (e.g., at home, school, or work; with friends or relatives; in other activities), and negatively impacts directly on social, academic, or occupational functioning. Several symptoms must have been present before 12 years of age^1^ and be present for 6 months, with behavior not matching the developmental level of the child.^2,3^

### Prevalence

Attention deficit hyperactivity disorder is reported to have a worldwide pooled prevalence of 5.3% as noted by Polancyzk et al^4^ by conducting a comprehensive systematic review.^5^ It is five times more common in boys than in girls.^3^

### Etiology

Etiology is unclear and various studies have suggested that it is a complex neurobiological disorder caused by the interaction of genetic, biological, environmental, and psychosocial risk factors.

Brain imaging studies suggest that there is smaller total cerebral volume as well as reduced global and local activation of the basal ganglia and the anterior frontal lobe in patients with ADHD. These areas of the brain are involved with executive functions, including impulse control, organization and planning, sustained goal-directed activities, and socially responsive behavior.^6^

Family studies have shown a higher occurrence among relatives of people with ADHD, with first-degree relatives having a four- to five-fold greater risk than the general population. A large-scale twin study by Levy et al estimated ADHD to be 70 to 90% heritable.

Some of the environmental factors predisposing to ADHD are suboptimal or inconsistent parenting, brain injuries or infection, low birth-weight or preterm delivery, maternal cigarette smoking, alcohol or drug abuse, and environmental toxins, such as lead, pesticides, mercury, manganese, or polychlorinated biphenyl.^4^

### Subtypes

According to DSM-IV, there are three subtypes: Combined, primarily inattentive, and primarily hyperactive/ impulsive.^7^ The combined subtype is the most prevalent, and patients display symptoms consistent with both inattention and hyperactivity/impulsivity. The primarily inattentive subtype is the second most common and was formerly known as attention deficit disorder. The primarily hyperactive/impulsive subtype is frequently described in very young children, who may have the combined subtype but have not reached the age where inattention becomes evident. The fourth subtype added by DSM-V, inattentive (restrictive), where the criterion for inattentive is met but no more than two symptoms from hyperactive/ impulsive have been present for the last 6 months.

### Clinical Features

In general, ADHD symptoms (inattention, hyperactiv-ity, impulsivity) become noticeable in primary school. However, in half of ADHD cases, this disorder is identified before the age of 4, which led to the hypothesis that it is a developmental disorder.^8^ It is the most common behavioral disorder in school-aged children and persists into adolescence and adulthood in approximately 30% of cases.^9^ The potential comorbidities include developmental language disorders, anxiety, oppositional-defiant behaviors, fine motor and coordination difficulties, and specific learning disabilities. They also have deficits in short-term auditory memory with extreme difficulty to retain short brief instructions.^10^

### Diagnostic Assessment

Diagnosis of ADHD is based on extensive assessment, which includes a detailed history (related to development, academic background and behavior), neurological, neuro-developmental, and physical examination and obtaining detailed behavioral rating as per the Conners scale from at least two sources.^11^ Most of the clinicians also use the diagnostic criteria of the DSM-IV.^1^ This stipulates that a diagnosis of ADHD can only be made if the child exhibits six of the nine defined symptoms in one or both categories of inattention or hyperactivity/impulsivity (Table 1). These must have been present since below age 7, are observed in at least two settings (i.e., school and home), and have persisted for at least 6 months (to exclude adjustment reactions to environmental stressors, such as parental separation, change of school, death of grandparent, etc.). In addition, the behavior exhibited by the child must be maladaptive (i.e., causing social, academic, or functional impairment) and be present to a developmentally inappropriate degree. Finally, in order for the symptoms (behaviors) to be ascribed to ADHD, other psychiatric disorders, such as autism and psychosis need to be excluded.^10^ New criteria for diagnosis were given in 2013, i.e., DSM-V.

### Dental Implications of ADHD

These children exhibit higher prevalence of dental caries,^12^ higher risk of molar-incisor hypoplasia, and are more prone to dental traumatic injuries.^4^ Higher prevalence of caries is due to poor oral hygiene practices as these children are forgetful and unable to brush the teeth effectively.

**Table 1:** **Table 1:** Summary of the DSM-IV (1994) diagnostic criteria for ADHD^10,11^

*Inattention*	
1. Often fails to give close attention to details in school work, work, or other activities	
2. Often has difficulty sustaining attention in tasks or play activities	
3. Often does not seem to listen when spoken to directly	
4. Often does not follow through on instructions and fails to finish schoolwork, chores, or duties in the workplace	
5. Often has difficulty organizing tasks or activities	
6. Often avoids, dislikes, or is reluctant to engage in tasks or activities	
7. Is often easily distracted by extraneous stimuli	
8. Is often forgetful in daily activities	
*Hyperactivity/Impulsivity*	
1. Often fidgets with hands or feet and squirms in seat	
2. Often leaves seat in classroom or in other situations in which remaining seated is expected	
3. Often runs about or climbs excessively in situations where it is inappropriate (in adolescents or adults may be limited to feelings of restlessness)	
4. Often has difficulty playing or engaging in leisure activities quietly	
5. Often “on the go” or acts as if “driven by a motor”	
6. Often talks excessively	
7. Often blurts out answers to questions before the questions have been completed	
8. Often has difficulty waiting in line or awaiting turn in games or group situations	
9. Often interrupts or intrudes on others (e.g., butts into conversations or games)	

Lalloo concluded that hyperactive children were twice as likely to experience an injury to the face and/or teeth as compared with the control group based on data from the 1997 Health Survey of England. This can be attributed to their involvement in violence resulting from conduct disorder. They have high plaque indices and low unstimulated salivary flow. Nail-biting and bruxism are commonly reported habits in these children and this was supported by a study done. They are also more likely to suffer physical abuse from a parent than a child in the general population.^4^

### Treatment Modalities

Management of the child with ADHD involves four broad approaches:

- Behavior modification
- Educational (counseling)
- Pharmacological
- Lifestyle changes ^2^

### Medications

Psychostimulant medication is the main pharmacological therapy for ADHD. The two stimulants most commonly prescribed are methylphenidate (Ritalin) and dexamphet-amine, which acts by increasing dopamine and norepi-nephrine. These medications produce significant clinical improvements in approximately 75% of correctly diagnosed children. The primary clinical effects are reduced physical and cognitive impulsivity and improved sustained attention, with secondary effects of increased work output, reduced conflict with family members and peers, and often improved self-esteem over time. Onset of behavioral effect is usually noticeable within 30 to 60 minutes of ingestion.

Other medications sometimes used in ADHD include the antihypertensive clonidine, antidepressants (selec-five serotonin reuptake inhibitors, reversible monoamine oxidase inhibitors, and tricyclics), and occasionally neu-roleptics.^10,14^ These medications have significant side effects, which must be considered by the dental health team, such as xerostomia, loss of smell acuity, sinusitis, dysgeusia, sialadenitis, stomatitis, gingivitis, discolored tongue, bruxism, dysphagia, elevated blood pressure, and raised heart rate.^13,15,16^

### Pharmacological Behavior Management

Sedation can be considered in children who do not respond successfully to nonpharmacological behavior management. Dentists should consider that use of sedative drugs must be with caution as these children are on stimulant drugs, which may antagonize the sedative effect.^17,18^ Although some practitioners have documented failed sedations or the requirement of higher drug con-centrations,^19^ others have successfully sedated children with the disorder. Before prescription of these drugs, the dentist must consult child’s physician. For managing these children effectively, sedative drugs, such as demerol, pro-methazine, and nitrous oxide can be used.^20^ Use of general anesthesia (GA) has not been extensively documented. A prospective study comparing the use of GA for children with and without ADHD undergoing elective surgery showed that induction procedures can be extremely challenging in those with ADHD, and that there was an increase in maladaptive behavior postoperatively.^21^

### Nonpharmacological Behavior Management in the Dental Clinic

There is increased likelihood of raised anxiety level in the children and parent on visiting dental clinic. In a child with ADHD, this anxiety may manifest in overexcited behavior. Many parents worry about the effect of their child’s behavior on others including the dentists.^10^ For the successful management of these children, it is imperative to follow these:

- Preappointment preparation: Child should be prepared before the commencement of treatment by making him/her acquainted with the dental environment. This can be done by advising the parent to make preappointment visit to the dental clinic along with the child, which will help in allaying the anxiety levels.
- Appointment should be scheduled early in the morning, because the child and dentist both will be less fatigued, child will be more attentive, and best able to remain seated on the chair. This time is also favorable attributing to the medicine peak effect. Appointments should be avoided during rebound periods.^18^
- Multiple short visits have a higher chance of success than a few, prolonged visits.^14^
- Use of frequent breaks thereby allowing child to indulge in his/her favorite activity has proved to be beneficial.
- Clear and simple instructions should be given repeatedly. Repetition is important in building up self-confidence in the child. Use of bright and colorful educational material has proved to be beneficial in imparting oral health education.^4^
- The use of the Tell-Show-Do method^4^ of behavior has been shown to have tremendous value in the management of children with ADHD. Praise and encouragement play an important role in the management of these children and good behavior should be reinforced and rewarded (positive reinforcement).^10^
- Use of protective stabilization (physical restraints) can be advocated in cases where children are not responding to other behavior management techniques.

### Special Care

Parental supervision is a must while performing oral hygiene procedures and also for diet control as both require concentration, motivation, and understanding, all of which can be a matter of issue for the child with ADHD. Anticipatory guidance^13,18^ should be provided to parents on how to prevent and manage dental injuries, as these children are prone to the same.

Home care instructions should be given in a written format, as these children are extremely forgetful and disorganized. It is also advisory to maintain tooth-brushing charts, to keep a record of practicing oral hygiene measures adequately at home.

Custom-fabricated occlusal splints^18^ is recommended for the treatment of bruxism. Syrups should be substituted with capsules or tablets as soon as the child is able to swallow them to prevent the chances of decay.

These children have been reported to be involved in substance use disorder. Documentation of the latter is highly significant as there can be drug interactions with conscious sedation and local anesthesia.^22-24^

**Fig. 1:**
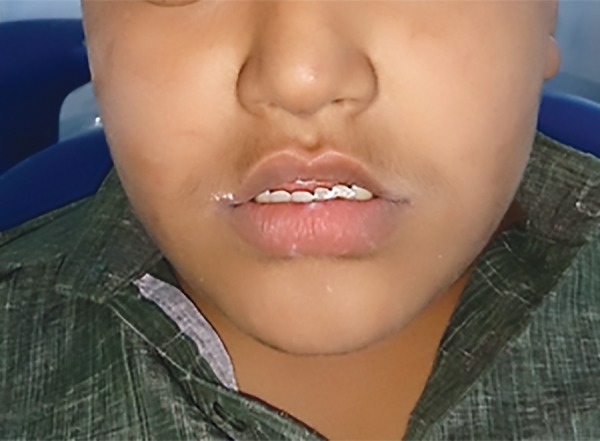
Extraoral photograph

**Fig. 2:**
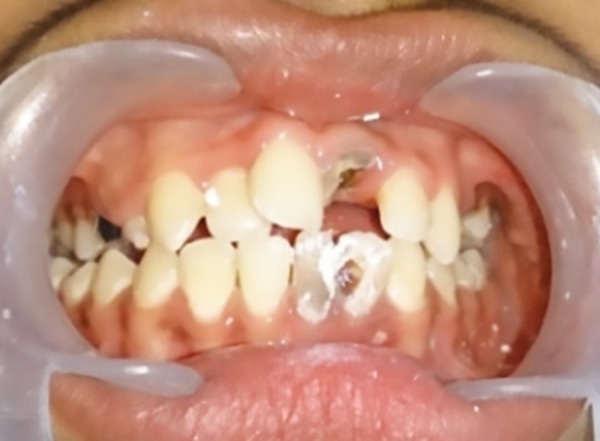
Preoperative intraoral photograph

**Fig. 3:**
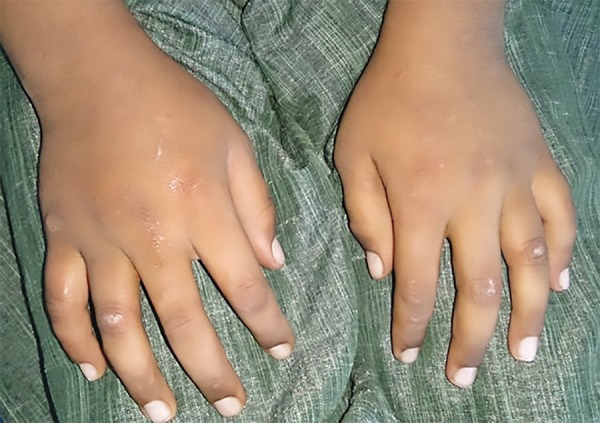
Marks present on the hands due to self-inflicted injuries

**Fig. 4:**
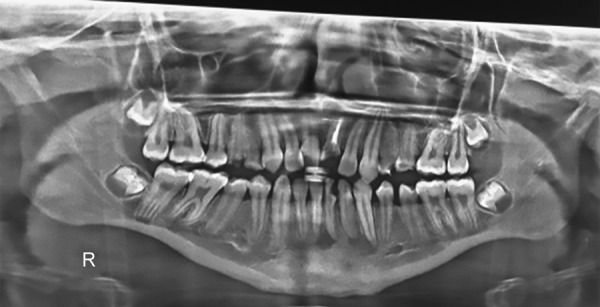
Preoperative OPG

Preventive programs and continuous reinforcement should be emphasized to minimize the need for complex restorative treatment. However, it is again imperative to realize that many of these children are already struggling to master other life skills.^10^

## CASE REPORT

A 14-year-old male child diagnosed with ADHD presented to the Department of Pedodontics and Preventive Dentistry at DA Pandu Memorial RV Dental College, with the chief complaint of broken tooth in upper front region since 2 years (Figs 1 and 2). Patient gave a history of root canal treatment done on the same tooth 2 years back.

Patient was diagnosed with ADHD (primarily hyperactive subtype) when he was 6 years old and was on medications Sizodon (Risperidone). He was unable to speak in sentences, unable to understand most of the things, and often got aggressive outbursts. Medical history revealed that the child was hospitalized 10 months back due to typhoid and there was increased frequency of micturition. There was a history of delayed milestones. Family history was insignificant except for the fact that the mother experienced high caries rate. Child had a habit of keeping food in the mouth for prolonged period (food pouching) and self-inflicted injuries, such as hitting on wall and banging head. He exhibited endomorphic features. Extraoral examination revealed scar marks on the face and upper limbs (Fig. 3). On intraoral clinical examination, there was no abnormal soft tissue finding. However, there was crown fracture up to gingival third with 21, dental caries with 16, 15, 26, 36, 46, retained 53, deep dentinal caries with 14, 24, 25, 31, and 32, unilateral crossbite on right side, and rotated 11. The patient was advised for orthopantomogram (OPG) (Fig. 4). Pulp vitality tests showed negative results with 24, 31, 32. After meticulous clinical and radiographic examination, following diagnosis was given: Root canal treated and fractured 21; chronic irreversible pulpitis with respect to 24, 31, 32; deep dentinal caries with respect to 14, 25; dental caries with respect to 16, 15, 26, 36, 46; retained 53; unilateral posterior cross-bite; and rotated 11.

**Fig. 5:**
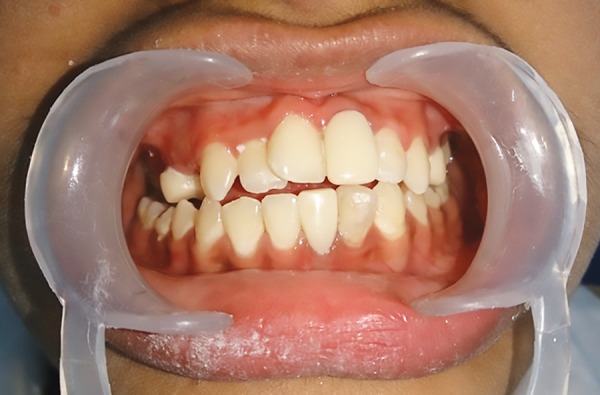
Postoperative intraoral photograph

**Fig. 6:**
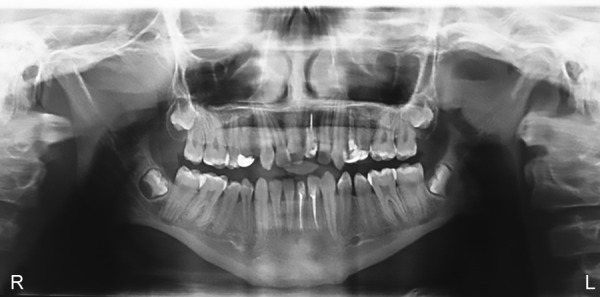
Postoperative OPG

Therefore, the treatment plan was oral prophylaxis, restoration with respect to 16, 15, 26, 36, 46, root canal therapy with respect to 24, 31, 32, core build-up with crown 14, 25, post and core with crown with respect to 21 (after gingivectomy), and extraction of 53. Orthodontic treatment was questionable due to child’s habit of self-inflicting injuries (Figs 5 and 6).

Child was reasonably cooperative during most of the treatment but turned extremely fidgety and slightly aggressive on few appointments due to skipping of doses of his medication. Although we had an option of treating the child under GA, but using appropriate nonpharma-cological behavior management, such as Tell-Show-Do, positive reinforcement, and breaks/time-outs, treatment was rendered successfully to the patient.

Patient came for a follow-up after 6 months, and on intraoral examination, he was caries-free and was maintaining oral hygiene properly. On a follow-up after a year, child had lost the acrylic crown on upper front tooth for which composite build-up was done.

## DISCUSSION

The ADHD patients present a unique challenge to the pedodontic team. However, with careful evaluation of their treatment needs and cooperative ability, they can be satisfactorily treated. This child presented with all the classical oral health issues reported in the literature. However, his age and ability to cooperate enabled us to treat him satisfactorily without the use of any pharmaco-logic methods. Few episodes of uncooperativeness were associated with not taking the prescribed medication. Thus, it is important to ensure the child has taken the medication before initiating dental treatment. This child also presented with an increased risk for trauma and self-injurious behavior, both of which was evident in this child. He was at high caries risk, due to combined effects of inability to practice adequate oral hygiene measures, habit of food pouching and medication (Risperidone) causing xerostomia. Usually children with ADHD exhibit molar-incisor hypomineralization, contrary to this case where child had hypoplastic premolars. Orthodontic treatment was highly questionable as the child already had a very compromised state of oral hygiene, and fixed mechanotherapy definitely demands for an exceptional cleanliness of the oral cavity. Frequent plaque controlling measures were instituted at weekly visits and proved to be effective.

The general ability to cooperate often results in a not-too-frequent use of GA in these children, as was our experience in treating this child.

For increasing the attention span over a period of time, these children should be encouraged to indulge in activities that require significant amount of concentration, such as video-games and tasks to arrange the colored blocks in a specific order. Similarly, candies can be used instead of blocks in younger children who are more likely to swallow the latter.

## CONCLUSION

Thus, with our clinical experience we would like to conclude that the dentists in general and pedodontists, in particular, should be knowledgeable to be in the forefront to make accurate diagnosis, use appropriate behavior management techniques, render prompt treatment with recurrent follow-up of these ADHD patients, and also to be able to make referral to the pediatrician and neurologists for promotion of good general and oral health of the patient.
